# Bamboo Classification Using WorldView-2 Imagery of Giant Panda Habitat in a Large Shaded Area in Wolong, Sichuan Province, China

**DOI:** 10.3390/s16111957

**Published:** 2016-11-22

**Authors:** Yunwei Tang, Linhai Jing, Hui Li, Qingjie Liu, Qi Yan, Xiuxia Li

**Affiliations:** 1 Key Laboratory of Digital Earth Sciences, Institute of Remote Sensing and Digital Earth, Chinese Academy of Sciences, No. 9 Dengzhuang South Road, Beijing 100094, China; lihui@radi.ac.cn (H.L.); liuqj@radi.ac.cn (Q.L.); yanqi@radi.ac.cn (Q.Y.); 2 School of Geography, Beijing Normal University, No. 19 Xinjiekou Wai Street, Beijing 100875, China; lixixi@mail.bnu.edu.cn

**Keywords:** classification, object-based analysis, bamboo mapping, WorldView-2, geostatistics

## Abstract

This study explores the ability of WorldView-2 (WV-2) imagery for bamboo mapping in a mountainous region in Sichuan Province, China. A large area of this place is covered by shadows in the image, and only a few sampled points derived were useful. In order to identify bamboos based on sparse training data, the sample size was expanded according to the reflectance of multispectral bands selected using the principal component analysis (PCA). Then, class separability based on the training data was calculated using a feature space optimization method to select the features for classification. Four regular object-based classification methods were applied based on both sets of training data. The results show that the *k*-nearest neighbor (*k*-NN) method produced the greatest accuracy. A geostatistically-weighted *k*-NN classifier, accounting for the spatial correlation between classes, was then applied to further increase the accuracy. It achieved 82.65% and 93.10% of the producer’s and user’s accuracies respectively for the bamboo class. The canopy densities were estimated to explain the result. This study demonstrates that the WV-2 image can be used to identify small patches of understory bamboos given limited known samples, and the resulting bamboo distribution facilitates the assessments of the habitats of giant pandas.

## 1. Introduction

As an endangered species, giant pandas (*Ailuropoda melanoleuca*) are threatened by continuous habitat loss and a low birth rate. Giant pandas live in a few mountain ranges in central China, mainly in Wolong, Sichuan Province, where bamboos act as the main food source for wild giant pandas. Estimating and mapping suitable habitat are critical to endangered species conservation planning and policy [1]. As a result, knowledge of the spatial distribution of bamboos becomes important for identifying the habitat of giant pandas. The increasing availability of remotely-sensed data has led to widespread use in habitat mapping. The common approach employed for habitat mapping using remote sensing is land cover classification, combined with ancillary information, such as digital elevation models (DEM) and the water system [2]. There have been ongoing studies for mapping bamboos and other tree species using remote sensing [3,4,5,6,7,8,9]. Most of these studies applied classification over large areas using medium or low spatial resolution imagery, such as Landsat TM/ETM+ [10,11,12,13,14] and MODIS [1], or using multi-temporal data, for example Wide Field Sensor data [3] and hyperspectral data [15].

In recent decades, the rapid development of satellite techniques has enabled researchers to work on tree species mapping using very high resolution (VHR) multispectral (MS) imagery [16]. Much research focused on extracting the desired land cover classes using VHR imagery. For example, Kamagata et al. [17] applied pixel-based and object-based classifications of IKONOS images to identify forest physiognomy. Ouma and Tateishi [18] estimated biomass by classifying QuickBird imagery. Pouteau et al. [19] also utilized QuickBird imagery to map rare plants. Sasaki et al. [20] classified tree species by integrating LiDAR and VHR imagery data. Hu et al. [21] explored the use of Google Earth imagery for land cover mapping in urban area. An accurate bamboo mapping can be realized with the increasing availability of VHR satellite imagery. For example, Araujo et al. [22] mapped bamboo-dominated gaps using QuickBird imagery. Han et al. [23] performed Moso bamboo forest mapping using SPOT-5 imagery.

WorldView-2 (WV-2), as a new satellite-borne sensor, was launched by DigitalGlobe Company in 2009. WV-2 is the first high resolution commercial satellite with eight MS bands. The data provider postulates that all four new bands (coastal blue, yellow, red edge and Near Infrared 2) are strongly related to vegetation properties [24]. Recent studies have also demonstrated that WV-2 imagery has a high potential in the classification of tree species. Immitzer et al. [25] examined the suitability of eight-band WV-2 satellite data for the identification of 10 tree species in Austria. Pu and Landry [26] explored the potential of WV-2 for identifying and mapping urban tree species/groups and compared capabilities between IKONOS and WV-2 imagery. Omer et al. [27] predicted the Leaf Area Index (LAI) of endangered tree species using WV-2 data. Karlson et al. [28] used WV-2 imagery to map tree crown in managed woodlands. Chuang and Shiu [29] used WV-2 pan-sharpened imagery to map tea crop. WV-2 has shown advantages in classifying bamboo patches, as well. For example, Ghosh and Joshi [16] compared classification algorithms for bamboo mapping using WV-2 imagery.

When processing VHR imagery, such as WV-2, advanced classification techniques are important, which have been studied for many years [30,31,32,33]. It is generally agreed that object-based image analysis (OBIA) is superior to pixel-based image analysis (PBIA) for processing VHR remotely-sensed data [34,35,36,37,38,39], because the former groups pixels into image objects (also known as segments), thus overcoming the salt and pepper effect, which often occurs in the latter. OBIA is now widely used to classify VHR images, mainly in land cover mapping of vegetation [32,40], forest [41], urban areas [26], shaded areas [42], burned areas [43], etc. For PBIA, much research has proven that spatial information can be used to improve classification results, such as geometry, homogeneity, entropy [44], contrast, dissimilarity [45,46], grey-level co-occurrence matrix (GLCM) and grey-level difference vector (GLDV) [47,48]. The OBIA approach also facilitates these features of image objects to be incorporated into classifiers. However, the spatial dependency (e.g., spatial correlation and heterogeneity) is rarely considered in OBIA. According to Tobler’s first law of geography, everything is related to everything else, but near things are more related than distant things [49]. Therefore, the spatial correlation between classes can be also incorporated to increase classification accuracy.

The Wolong natural reserve of giant pandas in Sichuan Province is a mountainous region. In this area, bamboos are sparsely distributed as fragments, mixed with brush and covered by tree canopies, thus causing difficulties in detecting and identifying bamboos. Therefore, this paper aims at exploring the possibility to accurately map small patches of understory bamboos using a VHR image in a complicated environment, which is critical to identifying habitats of giant pandas and supporting the conservation of endangered animals.

## 2. Materials and Methods

### 2.1. Study Area

The Wolong natural reserve is located in the southwest of Wenchuan County, Aba Tibetan and Qiang Autonomous Prefecture, Sichuan Province. This region is at the southeastern slope of Qionglai Mountains and is 130 km away from the provincial capital Chengdu City. In Wolong region, the habitat of wild pandas has been greatly shrunken and fragmented, due to agricultural expansions, increasing demands for timber products and infrastructure constructions. After the Wenchuan Earthquake happened in 2008, landslides and mudslides have worsened the situation.

There is a high variation in topography, soils and climate that leads to a diverse flora and fauna in Wolong reserve. Broadleaf forests are dominated by evergreen species below 1600 m and by a mixture of evergreen and deciduous species between 1600 m and 2000 m. Above 2000 m, a mixed coniferous and deciduous broadleaf forest gradually changes to a subalpine coniferous forest around 2600 m. The forest reaches about 3600 m, until it is replaced by alpine meadows. Under forest canopies, evergreen bamboo species dominate the understory layer [50].

**Figure 1:**
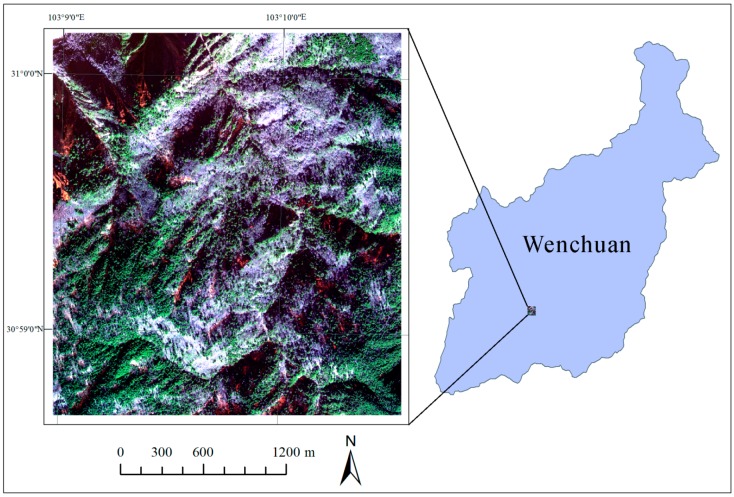
The study area in Wuyipeng (the true color composition of the WV-2 MS Bands 5, 3 and 2 as red, green and blue channels, respectively).

The study area is the Wuyipeng research site (Figure 1), which was once a research facility of the giant panda reserve center in Wolong, for researchers’ convenient access to the habitat of giant pandas. With the establishment of other giant panda reserves, this site is no longer fully in service. A WV-2 subscene (30°58′41″–31°0′10″ N, 103°8′57″–103°10′34″ E) with a size of 1383 × 1263 pixels, acquired on 14 January 2014 over the Wuyipeng area, is used in this study. The dataset consists of eight MS bands with a spatial resolution of 2 m, including coastal blue (0.400–0.450 μm), blue (0.450–0.510 μm), green (0.510–0.580 μm), yellow (0.585–0.625 μm), red (0.630–0.690 μm), red edge (0.705–0.745 μm), Near-Infrared 1 (NIR1) (0.770–0.895 μm) and Near-Infrared 2 (NIR2) (0.860–1.040 μm).

In the Wuyipeng area, there is an uphill route existing from northwest to southeast of the image, and the altitude changes from 1900 m–3450 m. There are mainly two bamboo species in this area: arrow bamboos (*Bashania fabri*) and umbrella bamboos (*Fargesia robusta*); and both of them are favored by giant pandas. Most umbrella bamboos grow taller than arrow bamboos, and both are sparsely distributed and covered by tree canopies at an altitude above 2000 m. However, the spectra of these two bamboo species are similar in the WV-2 imagery, and it is difficult to identify small patches of bamboo species using remote sensing techniques without hyperspectral information involved. Therefore, we did not identify tree species in this study, and the land cover types we focused on were classified as bamboo, coniferous, broadleaved, mixed woodland, brush and barren land.

### 2.2. Fieldwork

Extensive fieldwork at Wuyipeng was carried out in two field visits. The first one was on 11 June 2014, with the aim of measuring feature points for image geometric correction and collecting training data for classification. The second field visit was on 11–12 September 2014, for the purpose of testing the accuracy of the classification results. In both field trips, a Trimble^®^ GeoXH™ 6000 handheld GPS receiver (Trimble Mexico S. de R.L., Mexico City, Mexico) was used to collect sample points. An antenna was connected to the GPS with a 2-m centering rod to ensure that the GPS signals from multiple navigation satellites could be received under the canopy of large trees. The GeoXH handheld uses both EVEREST™ multipath rejection technology and H-Star™ technology to provide decimeter (10 cm) positioning accuracy. Finally, eight feature points were measured at the road junctions and the corners of houses, which were then used for geometric correction.

In the first fieldwork, about 300 points were sampled with their locations and vegetation categories recorded. However, since we did not know the precise locations of the image in the first fieldwork, many points fell into shadows after geometric correction, and their spectral information could not be used for training. Thus, these samples were discarded for classification. In the remaining training data, only three points were labelled as bamboos, and four for each of the remaining classes (coniferous, broadleaved, mixed woodland, brush and barren land). Commonly, the training and testing data should be sampled from different areas to make a fair comparison. However, only one route could be accessed to get to the top of the mountain, and there were no other routes in a different area nearby. There were still some small landslides in this area, making the place very dangerous to perform the fieldwork; therefore, the same area was explored in the second fieldwork, and 432 points were recorded as testing data. It should be noted that the shadow class was also used as a category for classification, which can be easily identified from the image. However, since it is not a typical land cover class type, the shadow class was not listed in the classification results.

### 2.3. Classification Methods

Several classification methods were used in the experiment, and a brief review of the related methods is presented in this section.

The Bayesian classification is based on Bayes’ theorem. It can predict class membership probabilities and then allocate a pixel to a class based on the maximum a posteriori decision rule.

The support vector machine (SVM) classifier is a supervised learning model with associated learning algorithms that analyze data and recognize patterns used for classification and regression analysis. Given a set of training data, each marked as belonging to one of two categories, an SVM training algorithm builds a model that assigns new data into one category or the other, making it a non-probabilistic binary linear classifier.

The classification and regression tree (CART) classifier is a non-parametric method, and its main idea is to recursively partition the data into smaller and smaller strata in order to improve the fit as best as possible. CART partitions the sample space into a set of rectangles and fits a regression model in each one. The sample space is originally split into two regions. The optimal split is found over all variables at all possible split points. For each of the two regions created, this process is repeated again. The major components of the CART method are the selection and stopping rules. The selection rule determines which stratification to perform at every stage, and the stopping rule determines the final strata to be formed. Once the strata are created, the impurity of each stratum is measured. The least squares model is used to measure the impurity of the nodes in regression trees. The heterogeneity of the outcome categories within a stratum is referred to as “node impurity”.

The *k*-nearest neighbor (*k*-NN) method classifies a pixel by majority voting according to its *k* neighbors in the feature space [51]. The geostatistically-weighted *k*-NN (g*k*-NN) classification was proposed by Atkinson and Naser [52] and was tested for the object-based method later [53]. In this method, the probability that a pixel *u* belongs to class *m* can be evaluated as follows:

(1) $$p_{gk-\mathrm{NN}}\left[c\left(u\right)=m\right]=\frac{\sum_{k=1}^{K}\left[S_{g}\times p_{m,m}\left(h_{uk}\right)\times \omega_{uk}+\left(1-S_{g}\right)\times \omega_{uk}\right]}{\sum_{m^{\prime }=1}^{M}\sum_{k=1}^{K}\left[S_{g}\times p_{m,m^{\prime }}\left(h_{uk}\right)\times \omega_{uk}+\left(1-S_{g}\right)\times \omega_{uk}\right]}$$

where **h** is the separation lag; the subscript *uk* of **h** indicates the lag between pixel *u* and its neighbor *k*. $p_{m,m}\left(h_{uk}\right)$ is the fitted model of the spatial covariance, which also refers to the class-conditional probability. *m′* is a class index for *m′* = 1, …, *M* classes, and *m* is the class of interest. *S_g_* is a proportional weight between 0 and 1. The larger the *S_g_*, the larger the weight given to the geographical component feeding into the probability. The class-conditional probability $p_{m,m^{\prime }}\left(h_{uk}\right)$ of a pixel *u* belonging to class *m*, given a neighbor *k* in class *m′* at a given lag **h**, is estimated as follows:

(2) $$p_{m,m^{\prime }}\left(h_{uk}\right)=\frac{\sum_{i=1}^{N}I\left[c\left(h\right)=m^{\prime }|c\left(u\right)=m\right]}{\sum_{i=1}^{N}I\left[c\left(u\right)=m\right]}$$

where *N* is the number of training pixels in the image, and *c*(**h**) represents the class value at a lag **h** (i.e., the class at the neighboring pixel location *k*). *I* is an indicator function. The function *I* takes a value of one if the condition is satisfied, otherwise zero. The spherical, exponential and Gaussian models are usually fitted to the class-conditional probability plot. The g*k*-NN method can account for the spatial dependence between the unknown location and its nearest neighboring (in the feature space) sample locations. Therefore, both spectral and spatial information iteratively affect the classification result.

## 3. Data Processing

**Figure 2:**
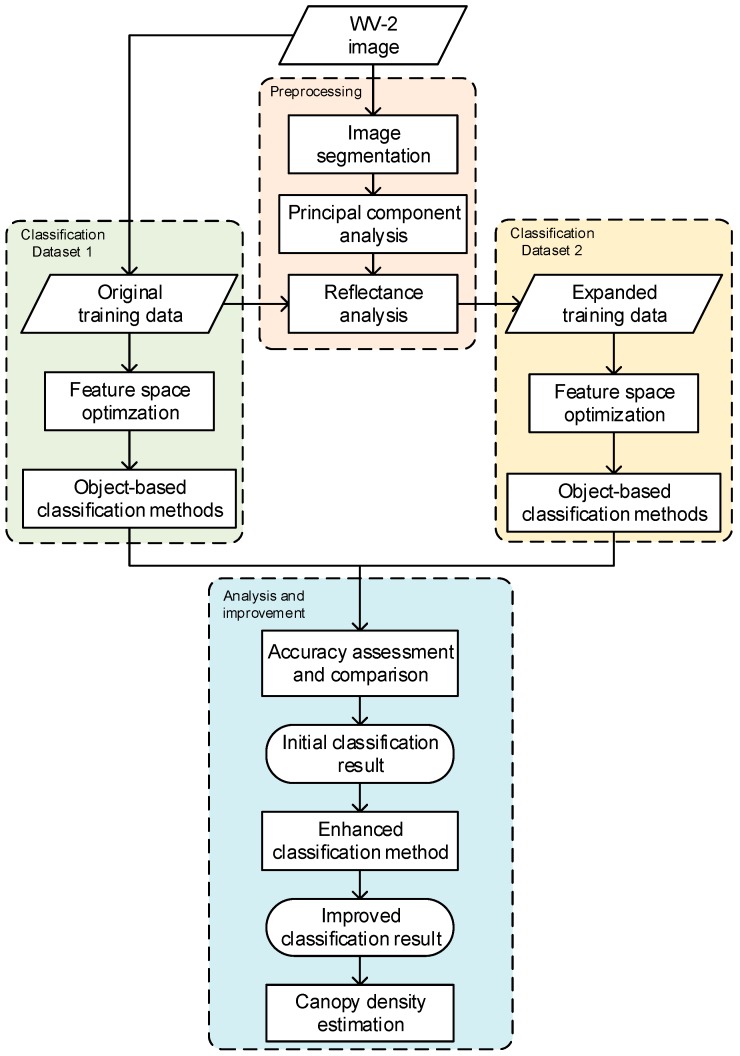
Flowchart of the classification process.

The OBIA classification scheme was adopted given such a high spatial resolution image, so the salt and pepper effect can be avoid in the classification. Furthermore, OBIA facilitates the incorporation of the geometry, texture and contextual information, which may be beneficial to classification. The flowchart is shown in Figure 2.

A multi-resolution image segmentation was first applied to the WV-2 image of all the eight MS bands. Then, 63,356 image segments were delineated given a scale parameter of 10, with a mean size of 27.6 pixels. The segmentation result was not further edited since it is difficult to visually identify boundaries in the image. However, the size of the training data (i.e., 23) sampled in the field is rather small relative to the total number of the image objects (i.e., 63,356), which may severely suppress the classification accuracy; it is therefore necessary to expand the size of training data to achieve a high classification accuracy. Thus, the principal component analysis (PCA) was performed to initially select important features, and then, a reflectance analysis was used to seek a method to expand the training data. After deriving the expanded training data, a feature space optimization method was tested on two sets of the training data to further select the features for classification. Then the original and expanded training data were both involved for classification, in which several methods were used to test the abilities of different classifiers given both sets of training data. The most effective classification scheme was selected, and an enhanced classifier was applied to increase the accuracy. Finally, the canopy densities were estimated to further explain the result.

### 3.1. Principal Component Analysis

The PCA was applied to all of the MS bands, along with three geometry features (the ratio of length to width, border index and shape index) and eight contextual features extracted from the grey-level co-occurrence matrix (GLCM) (mean, standard deviation, homogeneity, contrast, dissimilarity, entropy, correlation and angular second moment). The aim of PCA was to select the appropriate features for expanding training data and for classification, as well. The statistics and the loadings of the resulting principal components (PCs) are shown in Table 1 and Table 2, respectively. Only the statistics of the first ten PCs are shown in the tables.

Table 1 suggests that the first four PCs are critical, achieving a cumulative proportion of 1.0. The standard deviation of the first PC (PC1) is almost four-times more than that of the second PC (PC2), and the proportion of the variance of PC1 accounts for 0.91. Table 2 shows that Bands 6, 7 and 8 have the largest loadings (only depending on the absolute value) for PC1, which correspond to the red edge, and NIR1 and NIR2 bands, respectively. Band 3, corresponding to the green band, has the largest loading for PC2, whereas GLCM contrast has the largest loading for the third PC (PC3).

### 3.2. Expand Sample Size

As mentioned before, the proportion of classes and distribution of features may not be properly reflected due to a small sample size. Therefore, the sample size needs to be expanded to reduce its effects on the classification result. Here, a reflectance analysis was performed to check the spectral distribution of different classes across different bands, seeking a method to expand the training data.

**Figure 3:**
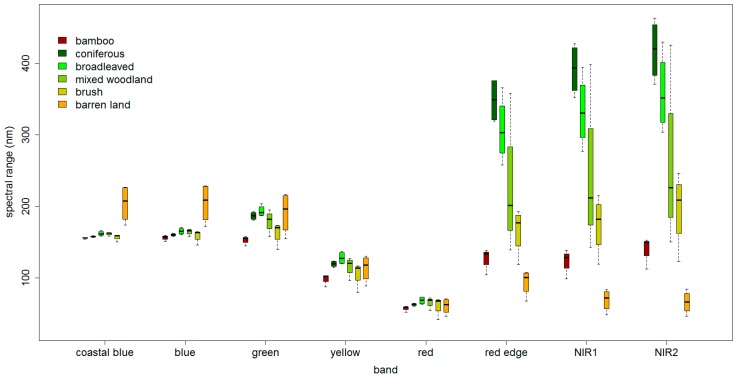
Distributions of the reflectance of different land cover types across eight MS bands.

According to the selected 23 training data, box-and-whisker plots of the spectral variability of the training data of six land cover types across eight MS bands are shown in Figure 3. The spectral reflectance is the mean value of the pixels within the segmented object. The bottom and top of the box are the first and third quartiles, respectively, and the band inside the box is the median value. As shown, the red edge and two NIR bands have stronger spectral separability of classes than other bands. The reflectance of bamboo is separable from all of the other classes across these three bands, but the spectral ranges of mixed woodland and brush overlap. The bamboo class also shows a great difference from other classes across the green and yellow bands. As indicated in Table 2, PC1 is exactly the combination of these five bands. Therefore, according to the reflectance analysis above, it is possible to utilize the mean value and the standard deviation of the reflectance for each class across these five bands to expand the training data.

**Figure 4:**
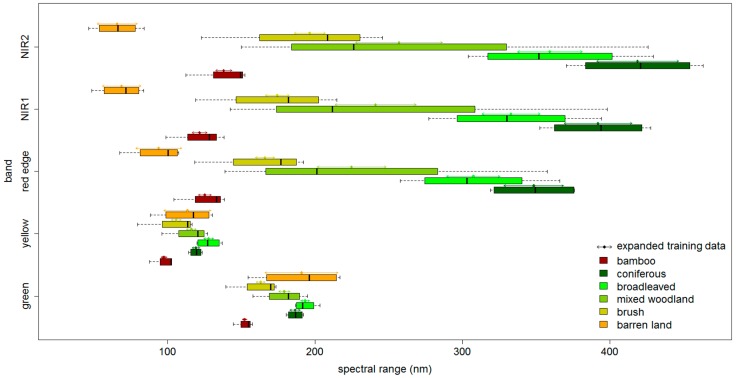
Distributions of the reflectance of different land cover types across five bands and expanded training data.

A parameter *t* is given for *µ* ± *tσ*, where *µ* and *σ* stand for the mean value and standard deviation of the reflectance, respectively. Two rules are followed: (i) the spectral range of each band is allowed little or no overlap between different classes; and (ii) an appropriate size of the expanded training data should be considered. Here, five MS bands of PC1 were considered for expanding the training data because the cumulative proportion of PC1 achieves above 90%. Another reason is that if too many features are included, the parameter *t* would take a large value in order to select enough training data, thus loosening the constraint of the features of training data. The parameters are shown in Table 3, and the spectral ranges of the expanded training data are indicated using arrows in Figure 4.

**Figure 5:**
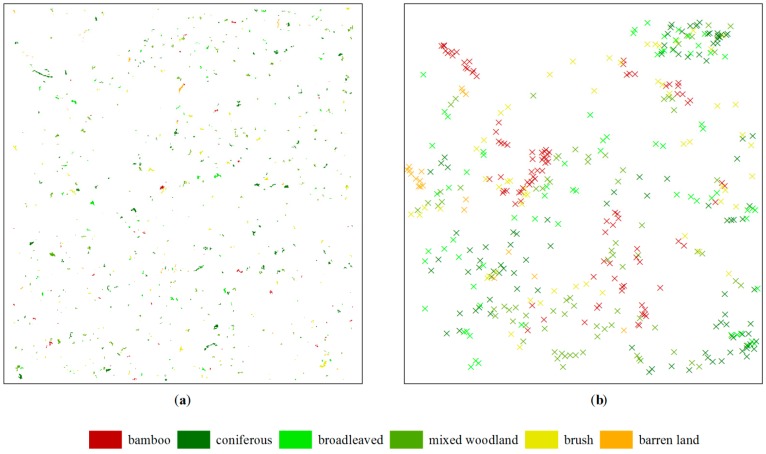
Spatial distribution of the samples: (**a**) training data and (**b**) testing data.

In Figure 4, the arrows show spectral ranges of the expanded training data for different classes across five bands. It can be seen that the red edge, NIR1 and NIR2 bands distinguish well all of the classes, but the spectral ranges of barren land and vegetation overlap in the green and yellow bands. After expanding the training data, as shown in Table 3, the total number of training data is 801 (including 83 samples of the shadow class), accounting for 1.26% of the total image objects (63,356). The spatial distributions of the expanded training data and the testing data collected in the second fieldwork are shown in Figure 5. The testing points are located within different segments to avoid redundancy of information, which may affect the reported accuracy.

### 3.3. Feature Space Optimization

It is common to use geometry and contextual features for object-based classification. However, the previous PCA result shows that only GLCM contrast has a great weight for PC3; the other geometry and contextual information do not contribute to the first four PCs. This is because there are rarely large vegetation patches in such a mountainous area; the geometry and contextual information do not show distinctive differences between small segments; thus this information cannot be effectively used.

**Figure 6:**
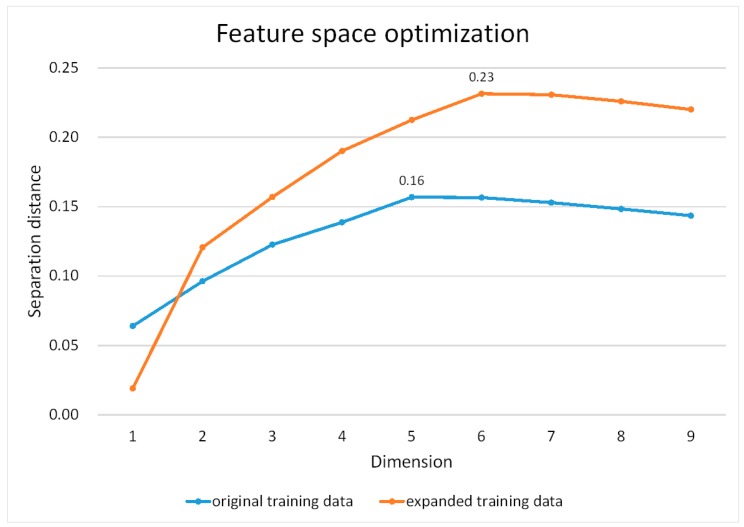
Feature space optimization using nine features based on two sets of training data.

Here, a feature space optimization method was used to further select the appropriate features for classification. We do not merely use the PCA selected features because PCA is estimated based on the whole image, whereas the class separability is also dependent on the features of training data. Therefore, nine features contributing to the first four PCs (the cumulative proportion achieves 1.0 using the first four PCs), including all eight MS bands and GLCM contrast, were used for feature space optimization based on two sets of training data. The barren land and shadow classes are easier to be identified, so these two classes were exclusive to avoid causing a dominated influence when estimating the optimized features. The method mathematically calculates the distances between the training samples of different classes in the feature space and chooses features that produce the largest average minimum distance as the best combination. The chart of the feature dimension against separation distance is shown in Figure 6. It turns out that five features (Bands 4–8) produced the largest distance (0.16) for the original training data, whereas the same five features and the GLCM contrast layer resulted in the largest distance (0.23) for the expanded training data.

In order to make a fair comparison using different training data, the same features should be involved for classification. Therefore, referring to the optimization result, we chose the six features selected based on the expanded training data. As a result, the yellow, red, red edge, NIR1 and NIR2 bands and a GLCM contrast layer were included for classification.

## 4. Results

### 4.1. Initial Classification Results

Four popular classification algorithms, including the CART, *k*-NN, Bayesian and SVM methods, were applied in this study. Both the original and expanded training data were used for classification based on the mean values of the yellow, red, red edge, NIR1 and NIR2 bands and the GLCM contrast layer.

**Figure 7:**
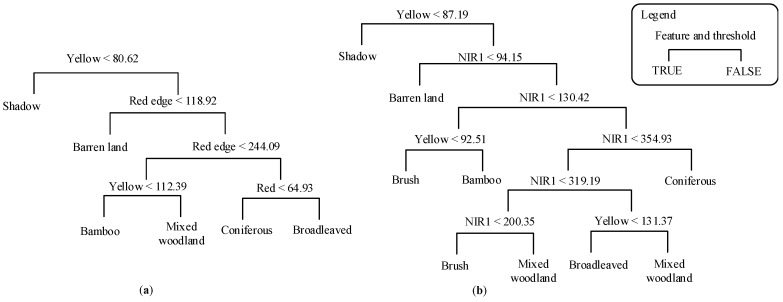
Decision rules of the CART classification based on (**a**) original training data and (**b**) expanded training data.

**Figure 8:**
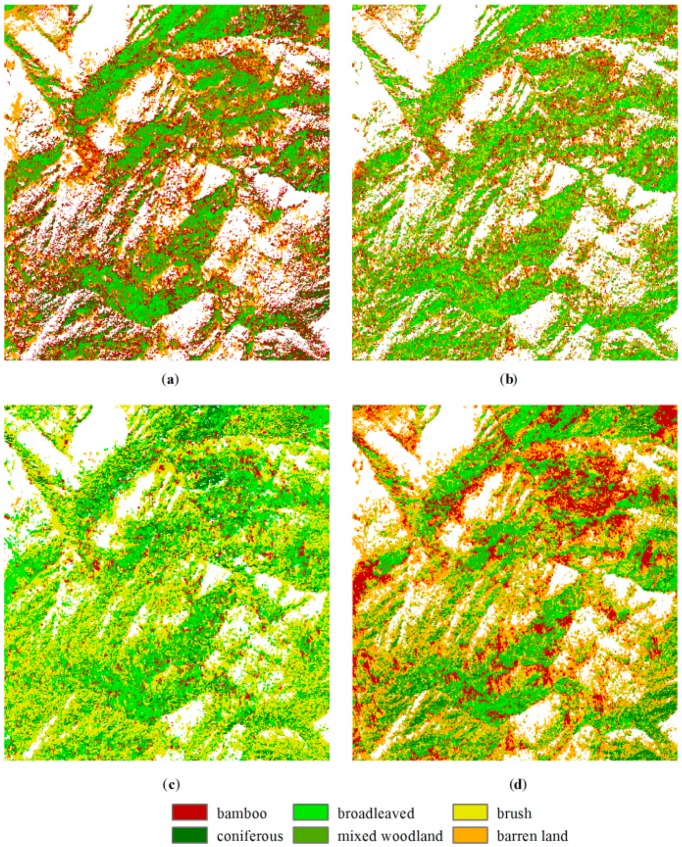
Classified maps generated using (**a**) CART; (**b**) *k*-NN; (**c**) Bayesian and (**d**) SVM methods based on the original training data.

**Figure 9:**
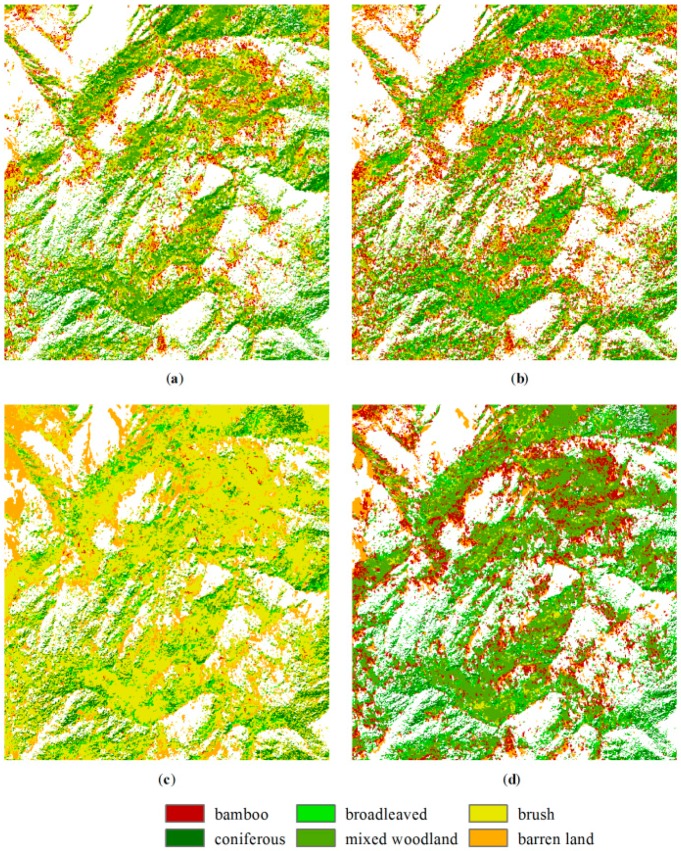
Classified maps generated using (**a**) CART; (**b**) *k*-NN; (**c**) Bayesian and (**d**) SVM methods based on the expanded training data.

In the CART method, the maximum depth of the decision tree was set to six; the minimum number of sample data for each node was set to five; and a six-fold cross-validation was performed. Figure 7 shows the decision rules of the regression tree. However, as can be seen, the original training data only involved three MS bands, and the brush class was missed in the decision rule, whereas the expanded training data distinguished all of the classes, but only using two MS bands. In the *k*-NN method, the *k*-value was set to five. In the SVM methods, a five-fold cross-validation was applied to select the cost of constraints violation *C* and the kernel parameter *γ* for the radial basis function (RBF) kernel. The optimal parameters *C* = 1 and *γ* = 0.2 and *C* = 2 and *γ* = 0.33 were used for the original and expanded training data, respectively. The classification results are shown in Figure 8 and Figure 9.

Comparing Figure 8 and Figure 9, the classification results using the Bayesian method show the most distinctive difference based on both sets of training data. The misclassification was caused by the non-normal distribution of the features and the inaccurate estimation of the covariance matrix. For the rest of the methods, the brush was not classified using the CART method based on the original training data. The results based on the expanded training data generally show more brush than those based on the original training data. The CART and SVM methods classify more areas as bamboos, and the *k*-NN method allocates more pixels as broadleaved based on the original training data. Comparing the four classification methods based on the expanded training data in Figure 9, brush is dominated in the Bayesian result. The SVM result has the highest number of pixels classified as bamboos, whereas the Bayesian result has the least. The broadleaved and mixed woodland classes appear more in the SVM result, and the broadleaved class also appears more in the *k*-NN result.

### 4.2. Accuracy Assessment

**Figure 10:**
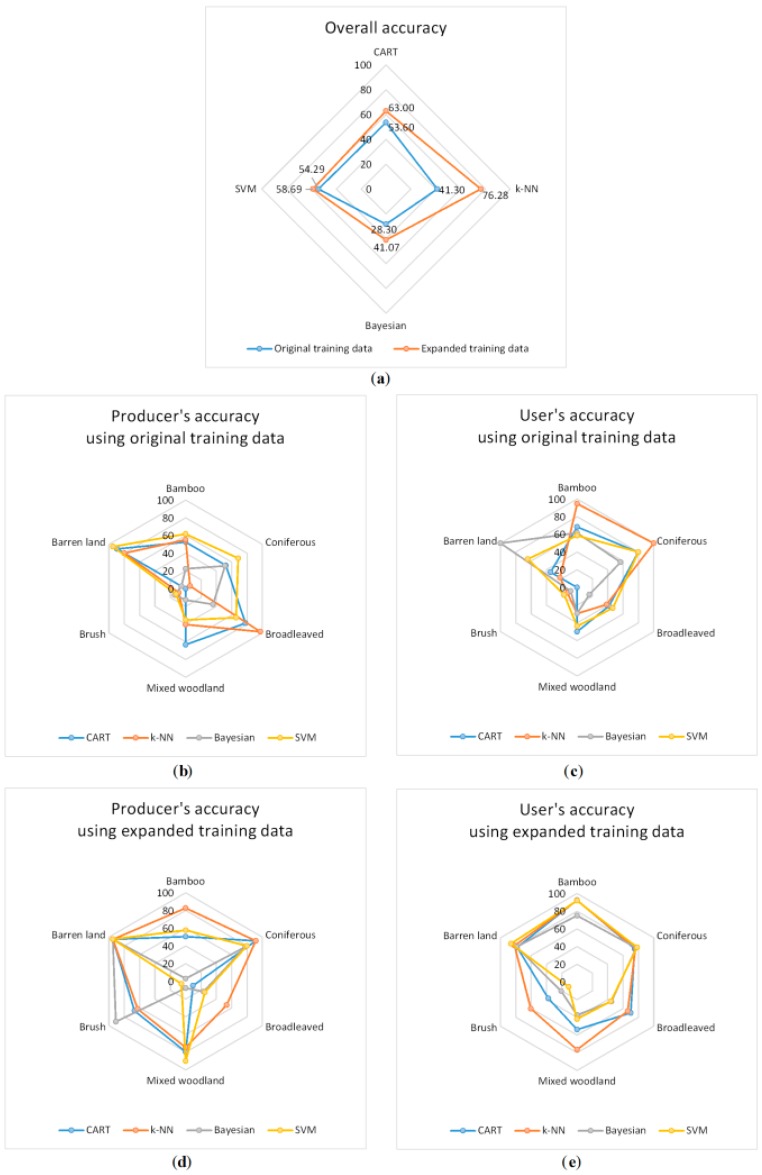
Radar charts of the accuracies using different classification methods based on the original and expanded training data (unit: %). (**a**) Overall accuracy; (**b**) Producer’s accuracy using original training data; (**c**) User’s accuracy using original training data; (**d**) Producer’s accuracy using expanded training data; (**e**) User’s accuracy using expanded training data.

The accuracies are reported as radar charts in Figure 10. As shown from Figure 10a, the overall accuracies are greater based on the expanded training data than for the original training data using all four classification methods. The SVM method has the greatest overall accuracy based on the original training data, but it only has a 4.4% increase in the overall accuracy, which indicates that the SVM is the most stable classifier; thus, sample size has little effect on it. The overall accuracies using the Bayesian method are the lowest based on both training data, but the increase in accuracy is great, which achieves 12.77%. The CART method increased 9.4% in accuracy based on the expanded training data, and its overall accuracy is greater than for the SVM method. The *k*-NN has the greatest overall accuracy based on the expanded training data, where the increase in accuracy is 34.98%.

Comparing the producer’s accuracies based on different training data, the SVM method shows a sharp decrease for the broadleaved class and an increase for the mixed woodland class based on the expanded training data. It reflects in Figure 8b and Figure 9b, where the dominant vegetation turned from broadleaved to mixed woodland. A similar situation also occurred for the CART method, where the dominant broadleaved class turned into brush, causing an increase in producer’s accuracy for the latter class. The classes of mixed woodland, brush and barren land suffer a severe misclassification based on the original training data using the Bayesian method; the brush and barren land classes were corrected based on the expanded training data, but the accuracy reduced for the bamboo class. The producer’s accuracies of all of the classes increased using the *k*-NN method, except for broadleaved forest, since it was classified more based on the original training data, as mentioned before. The user’s accuracies of all of the classes are generally greater based on the expanded training data than for the original training data, leading to an increase in the overall accuracy. Therefore, the expanded training data successfully increased the accuracies for all of the classification methods.

### 4.3. Improving the Classifier

Here, the *k*-NN method based on the expanded training data produced the most accurate result. In order to further increase the classification accuracy, a spatial weighting scheme was performed to improve the *k*-NN classifier and derive a contextual classification result. As described in Section 2.3, a geostatistical modelling was first performed to estimate class-conditional probability for each class in the g*k*-NN classification method and provided spatial information for the *k*-NN classifier. In the geostatistical model, the conditional probability is a function of distance. The Euclidean distance between segments was calculated to build the geostatistical model. In order to do so, the point located at the center of each segment was extracted using the FeatureToPoint tool (inside option) in ArcGIS. The central locations were recorded, and so, the distance between any two segments could be inferred.

**Figure 11:**
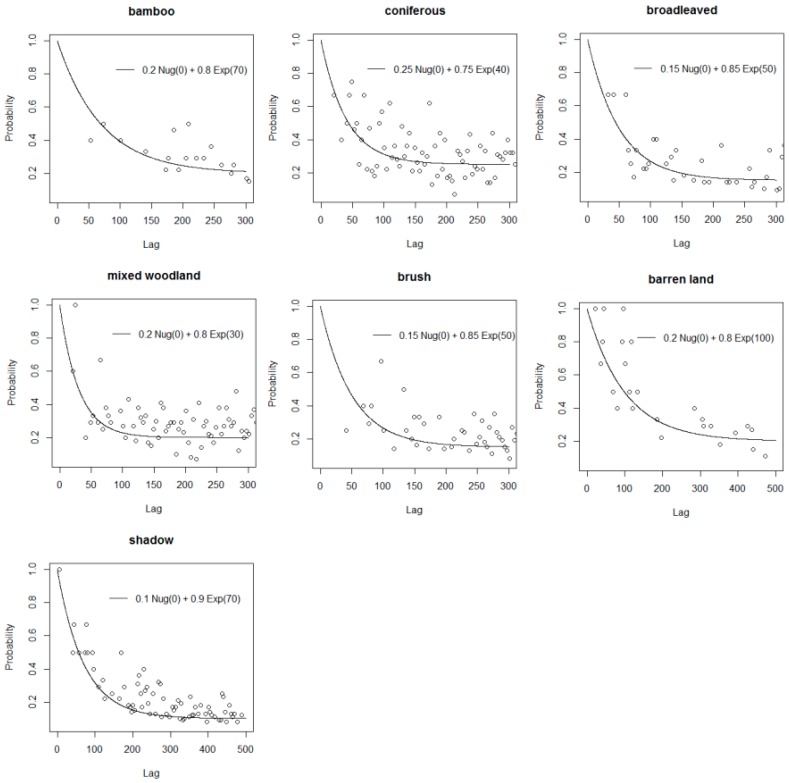
Estimated class-conditional probability plots and fitted models for each class. The lag on the *x*-axis is in units of pixels.

Class-conditional probability plots were estimated from 801 sample points and then fitted with the covariance models without considering anisotropy. The class-conditional probability plots for the seven classes and the fitted models are shown in Figure 11. Then, the g*k*-NN classification method using Equation (1) was performed, based on the same six features of the expanded training data as in the previous classification methods.

**Figure 12:**
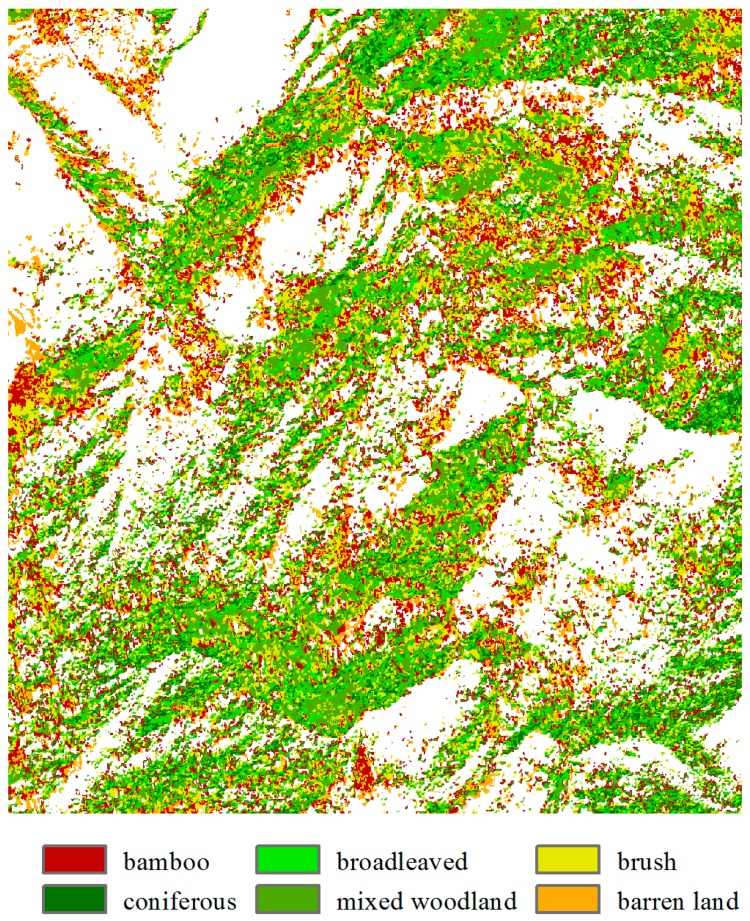
Classified map using the g*k*-NN method.

The classified map generated using the g*k*-NN method is shown in Figure 12. As indicated, Figure 9b and Figure 12 are similar in class distribution. The bamboo class in red color appears slightly more in the *k*-NN result than in the g*k*-NN result, whereas the mixed woodland class appears more in the g*k*-NN result. Table 4 and Table 5 show the complete confusion matrices and Kappa coefficients of the *k*-NN and g*k*-NN classification results. As can be seen, the overall accuracy and Kappa coefficient of the g*k*-NN method is 81.16% and 0.768, respectively, which are greater than for the *k*-NN method (76.28% and 0.706). The producer’s accuracies of the bamboo class are the same using both methods, and the user’s accuracy is slightly greater using the g*k*-NN method, indicating that the introduced spatial weighting has little effect on the bamboo class. The class that benefits the most from the spatial weighting is broadleaved forest, whose producer’s and user’s accuracies achieved the increases of 23.29% and 12.77%, respectively. The user’s accuracy of the coniferous class, the producer’s accuracy of the brush class and both the producer’s and user’s accuracies of the mixed woodland class increased. It can be seen in Figure 11 that as these four vegetation classes have shorter distance ranges than the other classes, the classification result was improved by spatial context within a short distance range. In conclusion, the spatial weighting used in the g*k*-NN classifier increased the accuracy of the *k*-NN method.

## 5. Discussion

### 5.1. Canopy Density Estimation

**Figure 13:**
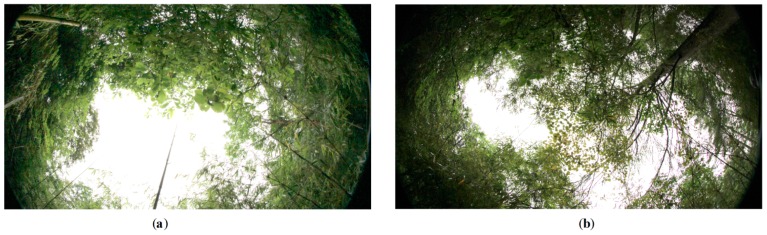
Tree crown photos taken using a fisheye camera at the testing locations. (**a**) The bamboo class surrounded by brush and was correctly classified; (**b**) the bamboo class covered by mixed woodland and was misclassified as brush.

In this study area, bamboos are covered by tree crowns at many locations, and their spectra do not show distinctive differences from surrounding vegetation and above tree crowns. Therefore, it is worth exploring what degree of canopy density (i.e., the percentage of vegetation to ground) can allow understory bamboos to be identified from the WV-2 imagery. Figure 13 shows two photos that were vertically taken using a fisheye camera at two testing points. Figure 13a was marked as the bamboo class surrounded by brush and was correctly classified, whereas Figure 13b was recorded as the bamboo class covered by mixed woodland, but was misclassified as brush using the g*k*-NN method.

**Figure 14:**
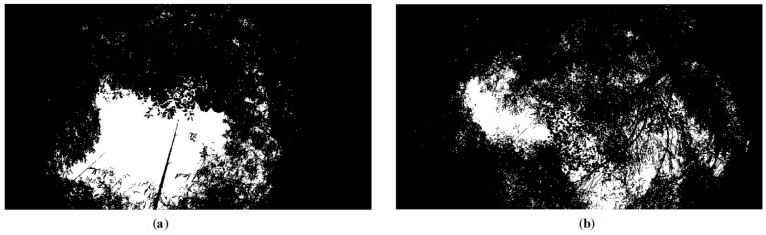
The canopies shown in binary maps of the photos shown in Figure 13. (**a**) The bamboo class surrounded by brush and was correctly classified; (**b**) the bamboo class covered by mixed woodland and was misclassified as brush.

It is obvious that the bamboos in Figure 13a are less covered by canopies than those in Figure 13b. The canopy densities were estimated for these two photos. Thus, the tree canopies were extracted and are shown in binary maps in Figure 14. The background pixels outside the fisheye camera were excluded by masking when estimating canopy densities. The canopy densities are 0.82 and 0.67 for Figure 14a,b, respectively. Therefore, in a WV-2 image, it is possible to extract bamboos in the areas with a median canopy density (from 0.2–0.7) or in a sparse forest (the canopy density is less than 0.2), but it is difficult to identify bamboos in the areas with a high canopy density (over 0.70).

### 5.2. Classification Accuracy Comparison

For the classification methods used in the experiment, the overall accuracies are all below 55% based on the original training data. This is because the classification model can be very vulnerable given such a small sample size, causing some class types to be severely misclassified, such as coniferous trees in the *k*-NN and brush in the CART methods. The expanded training data provided a proper proportion of classes as the prior knowledge and, therefore, increased the reliability of the classification model. The Bayesian classifier is sensitive to the distribution of features, whereas the SVM method shows its robustness in classification. The feature space optimization method is estimated based on the class separation distance, which is more suited to the *k*-NN method. This may be the reason that *k*-NN can achieve the greatest accuracy. However, even based on the expanded training data, the overall accuracies are not very high. There are three factors that may affect the accuracies: the understory bamboos, the diverse vegetation and some errors that have inevitably been introduced into the expanded training data, thus causing difficulties in classification. In order to compare the derived accuracy with other studies, Table 6 presents an overview of studies on bamboo classification carried out within the last 10 years using different remotely-sensed images.

The overall accuracies, reported in Table 6, range between 48% and 93%. However, the great accuracies are generally achieved in an area where bamboos are dominated and not covered by trees. For the classification of understory bamboos, the overall accuracies range between 74% and 88% and usually by using assistant data (e.g., elevation). The producer’s and user’s accuracies of bamboo obtained in this study (82.65% and 93.10%) are greater than most of the accuracies reported in comparable studies. Most of the methods for bamboo classification are widely used, such as the maximum likelihood classifier (MLC), SVM and some machine-learning methods, for example, neural networks and maximum entropy (MaxEnt). We also reviewed some advanced methods for classifying diverse vegetation (without bamboo class), including random forest [1], linear discriminant analysis [54,55], logistic regression [56], etc. However, it is more common to use hyperspectral images for forest classification [15,57,58,59,60,61]. The research using VHR images for bamboo classification has appeared very recently [16,23], and there is no related research using VHR images to extract understory bamboos as performed in this study. Elevation data and the derivatives can be combined to improve the classification results, which can be explored in the future. It should be noted that the objective of the paper was to check the possibility to extract understory bamboos using a WV-2 image in Wolong reserve rather than the validation of methods. Therefore, the methods in this paper including feature selection and classification are not universal; it may lead to different results when testing on another area.

### 5.3. Performance of the Gk-NN Method

It is worth exploring how the g*k*-NN method utilizes spatial information. Bamboos in the study area normally grow as small patches, and thus, unlike in previous research [16], texture information does not facilitate mapping bamboo patches. Instead, the spatial correlation was established between sparsely-distributed vegetated areas. The *k*-NN classifier was chosen to incorporate the spatial information because the location information of training data can be retained and a geographical weighting can be easily integrated into the classifier. It has been proven that both the distance weighted scheme and the geostatistical scheme can lead to sound classification results [52]. The spatial information lies in the conditional probability function, which derives from each pair of an unknown location and its nearest neighbors. For instance, the distance range of the covariance model for the bamboo class is 70 (Figure 11), which means that the spatial correlation between bamboos decreases within a distance range from 0–70 pixels (0–140 m), and the correlation will reduce to zero outside this range.

The last issue is the applicability of the g*k*-NN method in an object-based level. Although the classification applied in the case study is object based, the sample points for geostatistical modelling were obtained from the center of the training segments, and so, the covariance model describes the spatial correlation between different segments. The probability plots in Figure 11 show both the correlation within each segment (a peak) and the correlation between segments (a trend). The method characterizes the correlation between segments. Therefore, the peaks were ignored when fitting the geostatistical models. In this case study, as the number of segments is large and the average size of segments is small, the distances between centers of segments are similar to pixel-based measurement, and thus, the g*k*-NN method is applicable for the object-based classification.

## 6. Conclusions

This study explored the potential of VHR WV-2 imagery for extracting small patches of understory bamboos in a mountainous region in Wolong, Sichuan Province, China. A simple, but effective method was used to expand the training data to an appropriate sample size based on the PCA and reflectance analysis. The features of training data were then optimized for classification. Four regular object-based classification methods were applied based on both the original and expanded training data. The results were analyzed and compared through the field validation. The expanded training data successfully increased the accuracies for all of the classification methods, in which the *k*-NN method achieved the greatest accuracy. Then, an enhanced *k*-NN classifier weighted with a geostatistical scheme was applied to produce a final land cover map. This method produced the overall accuracy of 81.16%; the producer’s and user’s accuracies of the bamboo class are 82.65% and 93.10%, respectively. The canopy densities were estimated to check the possibility of extracting bamboos under tree crowns. This study demonstrates that the WV-2 image can be used to identify small patches of understory bamboos in a forest-covered mountainous area, given limited known sample points. The result is critical to identifying habitats of giant pandas and supporting the conservation of the endangered animals.

## Figures and Tables

**Table 1:** Importance of PCs.

	PC1	PC2	PC3	PC4	PC5	PC6	PC7	PC8	PC9	PC10
Standard deviation	188	50	22	15	10	6	4	2	2	1
Proportion of variance	0.91	0.06	0.01	0.01	0	0	0	0	0	0
Cumulative proportion	0.91	0.98	0.99	1	1	1	1	1	1	1

**Table 2:** Loadings of PCs (grey-level co-occurrence matrix (GLCM) Layers 1–8 represent the mean, standard deviation, homogeneity, contrast, dissimilarity, entropy, correlation and angular second moment, respectively).

	PC1	PC2	PC3	PC4	PC5	PC6	PC7	PC8	PC9	PC10
Band 1		−0.20				0.47	0.43			
Band 2		−0.35		−0.14	−0.24	0.63		0.39	−0.74	
Band 3	−0.14	−0.54		−0.22			−0.46	−0.65	0.48	
Band 4	−0.12	−0.53	−0.11	0.11		−0.36	0.67	−0.11		
Band 5		−0.40	−0.14		−0.18	−0.44	−0.33	0.59	0.30	
Band 6	−0.48			0.32	0.77	0.14		0.20	−0.35	
Band 7	−0.57	0.23		−0.76		−0.10	0.16			
Band 8	−0.63	0.17		0.48	−0.55			−0.14		
Length/width										−0.14
Border index										
Shape index										
GLCM 1										−0.45
GLCM 2										−0.56
GLCM 3										
GLCM 4			0.97			−0.15				
GLCM 5										−0.52
GLCM 6										−0.43
GLCM 7										
GLCM 8										

**Table 3:** The parameters to control the spectral range and the numbers of expanded training data.

Class	*t*	Spectral Range (Green, Yellow, Red Edge, NIR1, NIR2)	Sample Size
Bamboo	0.25	(150.6, 154.0), (95.6, 99.9), (120.7, 129.9), (116.7, 126.9), (132.6, 143.7)	49
Coniferous	0.65	(183.0, 190.2), (116.6, 122.1), (328.0, 368.7),(368.7, 415.1), (390.8, 446.5)	212
Broadleaved	0.4	(190.2, 196.4), (124.5, 131.3), (289.1, 325.5),(313.1, 352.6), (337.3, 381.2)	103
Mixed woodland	0.25	(175.4, 183.2), (112.8, 119.6), (201.4, 248.1),(213.7, 268.5), (227.4, 286.5)	209
Brush	0.2	(160.0, 166.4), (102.4, 109.5), (159.5, 172.7),(166.3, 182.6), (186.0, 206.9)	107
Barren land	0.85	(166.0, 215.7), (97.4, 129.7), (78.3, 109.7),(55.8, 82.1), (52.3, 79.6)	38

**Table 4:** Error matrix using the *k*-NN method (Class name: 1, bamboo; 2, coniferous; 3, broadleaved; 4, mixed woodland; 5, brush; 6, barren land), Kappa = 0.706.

*k*-NN	1	2	3	4	5	6	User’s Accuracy
1	81	0	0	0	7	0	92.05%
2	0	96	29	1	0	0	76.19%
3	1	2	39	11	6	0	66.10%
4	0	5	4	59	8	1	76.62%
5	13	2	1	7	35	0	60.34%
6	3	0	0	1	0	18	81.82%
Producer’s Accuracy	82.65%	91.43%	53.42%	74.68%	62.50%	94.74%	76.28%

**Table 5:** Error matrix using the g*k*-NN method (Class name: 1, bamboo; 2, coniferous; 3, broadleaved; 4, mixed woodland; 5, brush; 6, barren land), Kappa = 0.768.

g*k*-NN	1	2	3	4	5	6	User’s Accuracy
1	81	0	0	0	6	0	93.10%
2	0	95	10	1	0	0	89.62%
3	0	3	56	7	5	0	78.87%
4	0	5	3	62	8	0	79.49%
5	14	2	4	8	37	1	56.06%
6	3	0	0	1	0	18	81.82%
Producer’s Accuracy	82.65%	90.48%	76.71%	78.48%	66.07%	94.74%	81.16%

**Table 6:** Results from bamboo classification using different remotely-sensed images and classification methods from the last 10 years (in chronological order).

Image	Assistant Data	Methods	Class Number	Bamboo Accuracy (%) (PA/UA) ^1^	Overall Accuracy (%)	Understory Bamboo	Reference
Landsat TM	-	ANN ^2^	3	65/85	80	Yes	[10]
Airborne hyperspectral image	-	SAM ^3^	1	60	60	No	[61]
Landsat ETM+	Elevation, temperature, rainfall	MLC	5	84/41	88	No	[4]
ASTER	Elevation	ANN	7	77/84	74	Yes	[6]
MODIS	Elevation	MaxEnt	2	Kappa 0.74	88	Yes	[1]
Landsat MSS, TM, ETM+	-	MLC	12	n.s. ^4^	74	Yes	[62]
Hyperion EO-1	-	ANN	8	89/87	81	No	[15]
Digital photograph	LiDAR	Decision tree	16	57/56	48	No	[20]
Landsat TM, MODIS	-	Matched filtering	5	85	93	No	[9]
Landsat TM, MODIS	-	Unmixing	7	80/77	86	No	[63]
Landsat 8 OLI	Elevation	BPNN ^5^	12	84/n.s.	87	No	[64]
SPOT-5	-	CART	7	93/90	85	No	[23]
WV-2	-	SVM	7	94/89	91	No	[16]
VSWIR	-	EMC ^6^	8	72/98	65	No	[65]

^1^ PA: producer’s accuracy; UA: user’s accuracy; ^2^ ANN: artificial neural networks; ^3^ SAM: spectral angle mapper; ^4^ n.s.: not specified; ^5^ BPNN: back-propagation neural networks; ^6^ EMC: endmember average root mean square error, minimum average spectral angle and count-based.
